# Effect of Differences in the Microbiome of *Cyp17a1*-Deficient Mice on Atherosclerotic Background

**DOI:** 10.3390/cells10061292

**Published:** 2021-05-23

**Authors:** Axel Künstner, Redouane Aherrahrou, Misa Hirose, Petra Bruse, Saleh Mohamed Ibrahim, Hauke Busch, Jeanette Erdmann, Zouhair Aherrahrou

**Affiliations:** 1 Medical Systems Biology Group, Lübeck Institute for Experimental Dermatology, University of Lübeck, 23562 Lübeck, Germany; axel.kuenstner@uni-luebeck.de (A.K.); hauke.busch@uni-luebeck.de (H.B.); 2 Institute for Cardiogenetics, University of Lübeck, 23562 Lübeck, Germany; ra2qy@virginia.edu (R.A.); petra.bruse@uni-luebeck.de (P.B.); jeanette.erdmann@uni-luebeck.de (J.E.); 3 Centre for Public Health Genomics, Department of Biomedical Engineering, University of Virginia, Charlottesville, VA 22908-0717, USA; 4 Lübeck Institute for Experimental Dermatology, University of Lübeck, 23562 Lübeck, Germany; misa.hirose@uksh.de (M.H.); saleh.ibrahim@uni-luebeck.de (S.M.I.); 5 College of Medicine and Sharjah Institute for Medical Research, University of Sharjah, Sharjah 27272, United Arab Emirates; 6 DZHK (German Centre for Cardiovascular Research), University Heart Centre Lübeck, 23562 Lübeck, Germany

**Keywords:** Cyp17a1, mouse, knockout, microbiota, obesity, disorders of sex development, coronary artery disease, myocardial infarction, atherosclerosis

## Abstract

CYP17A1 is a cytochrome P450 enzyme that has 17-alpha-hydroxylase and C17,20-lyase activities. *Cyp17a11* deficiency is associated with high body mass and visceral fat deposition in atherosclerotic female ApoE knockout (KO, d/d or −/−) mice. In the present study, we aimed to investigate the effects of diet and *Cyp17a1* genotype on the gut microbiome. Female *Cyp17a1* (d/d) × ApoE (d/d) (DKO) and ApoE (d/d) (controls) were fed either standard chow or a Western-type diet (WTD), and we demonstrated the effects of genetics and diet on the body mass of the mice and composition of their gut microbiome. We found a significantly lower alpha diversity after accounting for the ecological network structure in DKO mice and WTD-fed mice compared with chow-fed ApoE(d/d). Furthermore, we found a strong significant positive association of the *Firmicutes* vs. *Bacteroidota* ratio with body mass and the circulating total cholesterol and triglyceride concentrations of the mice when feeding the WTD, independent of the *Cyp17a1* genotype. Further pathway enrichment and network analyses revealed a substantial effect of *Cyp17a1* genotype on associated cardiovascular and obesity-related pathways involving aspartate and L-arginine. Future studies are required to validate these findings and further investigate the role of aspartate/L-arginine pathways in the obesity and body fat distribution in our mouse model.

## 1. Introduction

Coronary artery disease (CAD), secondary to atherosclerosis, is the most frequent cause of morbidity and mortality worldwide [1]. Large-scale genetic studies have successfully identified loci that are associated with CAD, many of which have unknown physiological roles. One of the commonly reported genomic loci, which has been shown to be associated with CAD in several independent genome-wide association studies, is located on chr10p24.32. The same locus is associated with myocardial infarction [2], hypertension [3,4,5,6,7,8], and arterial stiffness in patients with diabetes [9], as well as with the visceral and subcutaneous fat masses [10]. This locus harbors the *CYP17A1* gene.

The CYP17A1 enzyme plays a crucial role in the steroidogenic pathway that is responsible for the synthesis of glucocorticoids, mineralocorticoids, androgens, estrogens, and progestins. Mutations in this gene are associated with congenital adrenal hyperplasia (CAH), a rare inherited disorder that affects both sexes [11,12,13]. Children and adolescents with CAH are at a high risk of obesity, owing to 21-hydroxylase deficiency [14]. We have generated mice that are deficient in CYP17A1, and in contrast to the results of a previous study [15], our knockout (KO) mice survived and displayed disorders/differences in sex development (DSDs) [16]. The CYP17A1-deficient female mice exhibited high body mass and visceral/subcutaneous fat deposition [16]. High CYP17A1 expression in the visceral adipose tissue of obese individuals was also recently reported by Ronquillo and co-workers [17]. Thus, there is strong evidence for the role of CYP17A1 in obesity and fat deposition.

The importance of the microbiota in human health and disease is well established. Gut dysbiosis is associated with many metabolic diseases, including obesity and cardiovascular diseases [18,19,20,21,22]. Previous studies have also demonstrated an association between some CYP family members (CYP7A1 and CYP17A1), gut bacteria, and atherosclerosis [23,24,25,26]. Castaner et al. reviewed the evidence on the association between intestinal microbiota and obesity and, lastly, the gut microbiota profiles in obesity [27]. Stanislawski et al. published recently how the gut microbiota-obesity relationship varied across heterogeneous Western populations and suggested that gut microbiota phenotypes of obesity may differ with race/ethnicity [28]. Furthermore, gut microbiota correlates with dietary or socioeconomic status [28]. Gut bacteria have been documented as an important contributor to the development of atherosclerosis [29,30,31]. Sanchez-Rodriguez and co-workers reviewed the implication of gut microbiota in the development of atherosclerosis and related cardiovascular diseases and examined its beneficial aspect as a therapeutic target in the prevention of cardiovascular diseases [32].

We have provided evidence that CYP17A1 is associated with obesity and atherosclerosis in our previous study, but the relationship between CYP17A1 and the gut microbiota remains unclear. The Cyp17A1 heterozygous mice were backcrossed to the commonly used atherogenic ApoE (Apolipoprotein E) deficient genetic background to induce atherosclerosis and obesity when feeding a Western-type diet (WTD) [16].

In the present study, we aimed to determine the effects of diet and *Cyp17a1* genotype on the composition of the microbiota in a mouse model of atherosclerosis (*ApoE* KO).

## 2. Materials and Methods

### 2.1. Animal Feeding and Collection of Samples

This study was approved by the German Animal Studies Committee of Schleswig-Holstein and was conducted in compliance with international guidelines. Two groups of female mice, with genotypes *Cyp17a1*(d/d) × *ApoE*(d/d) and *ApoE*(d/d), were studied. The symbol “d” stands for deletion and is similar to KO or “−”. From the age of 10 weeks, mice were fed either a standard chow diet (chow) or a Western-type diet (WTD) containing 0.2% cholesterol and 21.2% fat (TD.88137; ssniff Spezialdiäten GmbH, Soest, Germany) for 8 weeks. The mice were maintained under controlled conditions of temperature (23 °C), humidity (40–60%), and lighting (12 h/12 h light/dark cycle). After 8 weeks of diet-feeding, the mice were euthanized using an overdose of isoflurane inhalation, followed by cervical dislocation, and then perfused with phosphate-buffered saline, pH 7.4 (Lonza, Cologne, Germany). Blood samples were collected to study the lipid profiles of the mice, as described previously [16]. The body mass and visceral fat mass were determined as previously described [16]. Finally, the caeca of the mice containing feces were collected, and tail biopsies were obtained for re-genotyping. All the samples collected were stored at −80 °C until further analysis.

### 2.2. DNA Isolation, PCR, and 16S rRNA Sequencing

Bacterial DNA isolation and sequencing were performed as reported previously [33]. Briefly, the cecal samples were collected, and DNA was isolated using a Power Soil DNA Isolation KIT (Qiagen, Hilden, Germany), according to the manufacturer’s protocol. Specific primers targeting the V1-V2 hypervariable region (27F-338R) of the bacterial 16S rRNA genes were used to amplify these sequences by PCR, using a similar reaction mix to that published previously [33]. PCR products were analyzed on 1.5% agarose gels and visualized using Sybr Green II, and then individual DNA bands were pooled to create approximately equimolar sub-pools and purified. These sub-pools were mixed in equimolar amounts and stored at −20 °C until sequencing, which was performed on the Illumina MiSeq platform using v3 chemistry.

### 2.3. Sequence Processing and Data Analysis

Demultiplexed raw sequencing data (*fastq* format) were processed into amplicon sequence variants (ASVs) using DADA2 (v1.18.0) [34]. Briefly, forward reads were trimmed to 280 bp and reverse reads to 260 bp, the expected error rate was assigned the value 2, and size selection was set to 280–343 bp. Furthermore, chimeric sequences were removed. Taxonomic assignment was performed using DECIPHER (v2.18.1) [35], with GTDB r89 [36] as the reference database. Potential contaminants were removed using the frequency method, as implemented in the R package decontam (v1.10.0) [37], with the threshold set to 0.1. Nine ASVs were identified as contaminants and removed. ASVs not belonging to the kingdom Bacteria or with unassigned phylum were excluded from further analysis.

### 2.4. Statistical Analysis

ASV data and covariates were imported into R (v4.0.3; https://cran.r-project.org/bin/windows/base/, accessed on 12 November 2020). Alpha diversity, based on non-normalized counts, was estimated using DivNet (v0.3.6; sample-wise and community-wise Shannon diversity) [38], and richness was estimated using breakaway (v4.7.2) [39]. Differences in alpha diversity were calculated using the *betta* function (breakaway v4.7.2) [40] to provide richness and sample-wise estimates and *testHypothesis* (DivNet) for community-wise estimates, respectively. To assess beta diversity, Aitchison distance was applied. Briefly, data were centered log-ratio transformed (clr), and distances were calculated using Euclidean distance. Permutational multivariate analysis of variance using distance matrices (PERMANOVA) was used to analyze differences in beta diversity (*adonis* function, as implemented in the vegan package v2.5-7, with 99,999 permutations). Differential abundance analysis was performed using analysis of compositions of microbiomes with bias correction (ANCOMBC package v1.0.2 [41]; 10,000 iterations, zero cut-off 0.9, Benjamini–Hochberg correction to adjust for multiple hypothesis testing). The R package microbiomeMarker (v0.01.9000) was used to perform linear discriminant analysis coupled with effect size (LEfSe) [42] using 1000 bootstrap iterations with correction for multiple testing, and the results were restricted to phylum and genus level.

To retrieve functional profiles for the ASV data, sequences and abundance tables were used as inputs for PICRUSt2 [43]. The results were visualized using Aitchison distance, and differentially abundant KEGG identifiers were identified using ALDEx2 (v1.22.0) [44]. The annotation of the KEGG identifiers was performed using KEGGREST (v1.30.1), and effect sizes were estimated using Hedge’s g statistic (dabestr v0.3.0). Enrichment analysis for Gene Ontology (GO) Biological Processes was performed using enrichR (v3.0).

Correlation network inference on clr-normalized abundances was performed using the SparCC [45] approach, as implemented in the R package NetCoMi (v1.0.2) [46], and significant edges were selected using Student’s *t*-test. Community structures were estimated using greedy optimization of modularity, hub node detection was performed using a threshold of 0.8, and quantitative assessment of the network was performed using a permutation approach (100,000 bootstraps) with an adaptive Benjamini–Hochberg correction to adjust *p*-values for multiple testing.

For linear regression analysis, *lm_robust* (estimatr package v0.30.2) was used. Visualization of the microbiome analyses were performed using ggplot2 (v3.3.3), ggpubr (v0.4.0), and patchwork (v1.1.1); the R packages phyloseq (v1.34.0) [47] and tidyverse (v1.3.0) were used for data handling.

## 3. Results

We have previously shown an association of *Cyp17a1* KO with obesity in female mice [16]. Here, we asked whether the *Cyp17a1* KO affects gut microbial diversity and composition.

### 3.1. Cyp17a1 KO Is Associated with High Body Mass and Fat Volume

**Figure 1:**
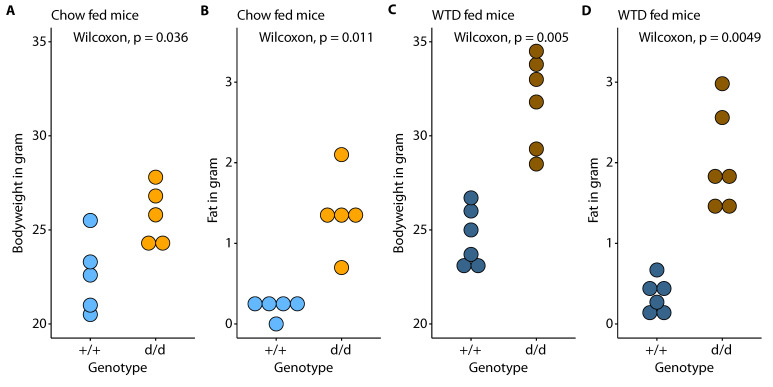
Characteristics of female Cypa17a1 control and knockout mice. Comparison between ApoE KO control (+/+) and double knockout (d/d) mice for bodyweight (**A**,**C**), and body fat (**B**,**D**) with respect to diet (chow and Western-type diet; WTD).

Female Cyp17a1 KO mice exhibited much higher body mass and fat volume than their wild-type (WT) littermates when chow-fed (Figure 1), and these differences were more marked after feeding the mice a WTD; characteristics of mice are presented in Table S1.

### 3.2. Microbial Diversity

**Figure 2:**
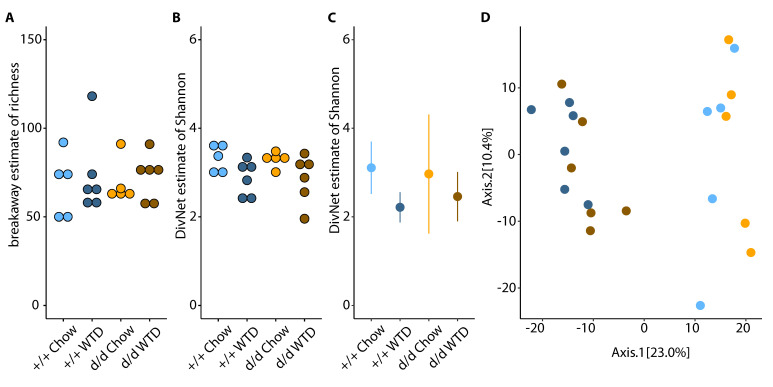
The effect of diet and genotype on diversity (alpha and beta). Estimated species richness and Shannon diversity (both alpha diversity measurements) are shown in panels (**A**,**B**) for each sample, grouped by genotype and diet. Panel (**C**) shows the community-wise estimate of Shannon diversity per genotype and diet. Beta diversity was estimated using Aitchison distance, and the results are shown in a principal coordinate analysis plot (**D**); light colors refer to controls (d/d) and dark colors to double knockouts (+/+); blue refers to chow-fed mice and orange to WTD-fed mice. Axis 1 (x-axis) explains 23.0% and Axis 2 (y-axis) 10.4% of the total variance, respectively.

A total of 22 female mice were studied (Control *ApoE*(d/d) mice: *n* = 5 chow and *n* = 6 WTD; double knockout (DKO) *Cyp17a1*(d/d) × *ApoE*(d/d): *n* = 5 chow and *n* = 6 WTD), which yielded 480 ASVs for further analysis. The number of contigs ranged between 4971 and 23,547 (mean: 14,219; SD 5166). Alpha diversity was assessed using species richness and the Shannon index. For species richness, no significant correlation with the number of contigs per sample was identified (F_1,20_ = 0.8536, R^2^_adj_ = 0.0226, *p* = 0.3665). Furthermore, no significant differences in species richness were identified (Figure 2A, betta test, *p* > 0.05) *versus ApoE*(d/d) controls consuming a chow diet. To further investigate alpha diversity, we estimated the Shannon index sample-wise, with the assumption that the taxa in a community form an ecological network [40]. Control WTD-fed mice and WTD DKO mice showed significantly lower Shannon diversity (*p* = 0.005, difference −0.4446, SE 0.1593 and *p* = 0.004, difference = −0.4546, SE 0.1593; respectively) than control chow-fed mice, whereas chow-fed DKO mice showed a similar Shannon diversity (*p* = 0.962) to chow-fed control mice (Figure 2B). Community-wise estimates of the Shannon index generated similar results (Figure 2C), with lower diversity in both WTD-fed groups (Control: *p* < 0.001, difference −0.8927; DKO: *p* < 0.001, difference −0.6500) and no difference between the chow KO group (*p* = 0.643) and chow-fed controls.

The bacterial composition of the four groups (beta diversity) was compared using Aitchison distance. We found a significant effect of diet on beta diversity (PERMANOVA, *p* = 1.0 × 10^−5^, R^2^ = 0.2193), which accounted for about 22% of the observed variation in bacterial composition, but no effect of genotype (*p* = 0.3112, R^2^ = 0.0414) and no interaction between genotype and diet (*p* = 0.2934, R^2^ = 0.0420). Projections of the first and second principal coordinates are shown in Figure 2D.

### 3.3. Abundance of Each Taxon

**Figure 3:**
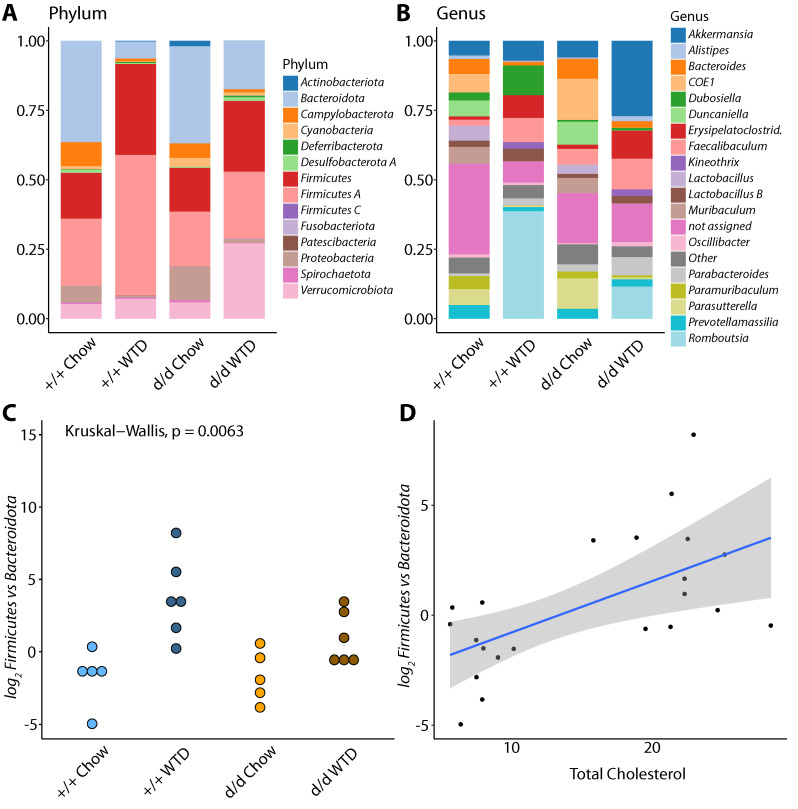
Taxonomic differences and Firmicutes vs. Bacteroidota ratio. Taxonomic composition for each genotype and feeding regime are shown for phylum (**A**) and genus (**B**) level; the y-axis shows the proportion of mapped contigs. For genus composition, the top 19 genera are shown, and the remaining genera were binned together into ‘Other’ (grey color). (**C**) shows the log2-scaled Firmicutes vs. Bacteroidota ratio per genotype and diet. The relationship of the log2-scaled Firmicutes vs. Bacteroidota ratio and measured total cholesterol is shown in (**D**); the blue line refers to the fit of the linear model, and the shaded area denotes the 95% confidence interval of the linear fit.

The mean abundances of all the phyla and the top 19 genera identified are shown in Figure 3A,B. To find out alterations associated with diet and genotype, linear discriminant analysis coupled with effect size (LEfSe) was performed, and the results were restricted to phylum and genus level, resulting in 11 features with LDA scores > 2.0 (*p_adj_* < 0.05). Control chow-fed mice showed an increase in the genus *Lactobacillus*, whereas control WTD mice showed higher abundances of the phyla Firmicutes, and Firmicutes A and the genus *Romboutsia*. Chow-fed KO mice showed higher abundances of the phylum Proteobacteria and the genera *CO1*, *Duncaniella*, *Lactobacillus H, Muribaculum,* and *Parasutterella;* WTD-fed KO mice showed enrichment in the genus *Kineothrix*. The results are shown in Table S2 and Figure S1.

Next, differential abundance analysis was performed using chow-fed control mice as a reference to investigate changes in composition due to diet and genotype using analysis of compositions of microbiomes with bias correction (ANCOMBC). Results showed that in comparison to control chow-fed mice, the phylum Bacteroidota was significantly less abundant (*p_adj_* = 0.0351) and the phylum Firmicutes A was significantly more abundant (*p_adj_* = 0.0351) in control WTD-fed mice; and the phylum Deferribacterota was significantly less abundant in chow-fed DKO mice (*p_adj_* = 0.0010). Some genera showed similar trends when control WTD-fed mice were compared with chow mice, regardless of their genotype. The genera with lower abundances in WTD-fed mice were *CAG-475*, *COE1*, *Duncaniella*, *Lactobacillus*, *Muribaculum*, and *Rs-D84* (*p_adj_* < 0.05), whereas *Faecalibaculum* and *Romboutsia* were significantly more abundant (*p_adj_* < 0.05) in these mice. None of the genera significantly differed in abundance between the DKO groups and WT chow-fed groups. All the phyla, families, and genera that were evaluated are listed in Table S3. To further evaluate the contribution of the identified differences in the abundance of phyla, the ratios of the abundances of *Firmicutes* and *Bacteroidota* (GTDB taxonomy; NCBI taxonomy name: *Bacteroidetes*) were calculated. There was a significant difference in this ratio among the four groups (Kruskal–Wallis test, *p* = 0.0063), and post hoc testing (pairwise Wilcoxon tests with Benjamini–Hochberg correction) revealed that the difference was due to diet, rather than genotype (Chow^+/+^ vs. WTD^+/+^ *p_adj_* = 0.026, Chow^+/+^ vs. WTD^d/d^ *p_adj_* = 0.035, Chow^d/d^ vs. WTD^+/+^ *p_adj_* = 0.026, all remaining comparisons *p_adj_* > 0.05, Figure 3C). Furthermore, there was a significant correlation between the ratio and the circulating total cholesterol concentration of the mice (F_1,20_ = 9.768, R^2^_adj_ = 0.3213, *p* = 0.0053; Figure 3D).

### 3.4. Functional Profiling

**Figure 4:**
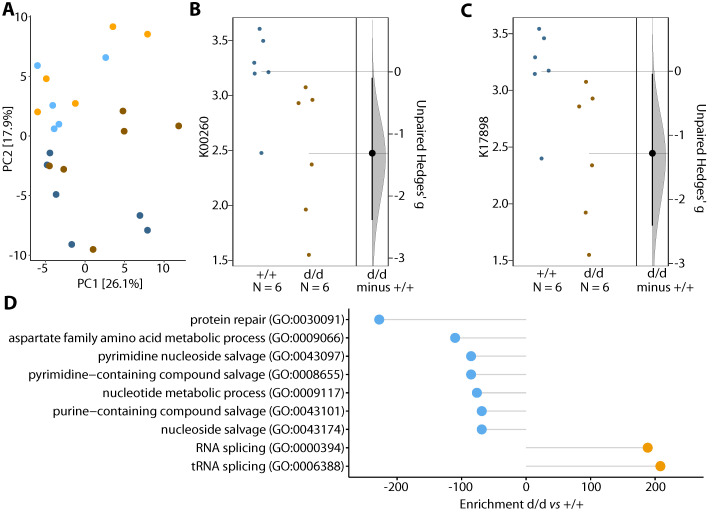
Functional profiling of the gut microbiome. Redundancy analysis plots of clr-transformed functional profiles (estimated by PICRUSt2) are shown in panel (**A**); light colors refer to controls (d/d) and dark colors to double knockouts (+/+); blue refers to chow-fed mice and orange to WTD-fed mice. Axis 1 (x-axis) explains 26.1% and Axis 2 (y-axis) 17.9% of the total variance observed, respectively. Gardner-Altman (effect size) plots for Arginine-related KEGG IDs for WTD-fed mice are shown in (**B**,**C**). Effect size (unpaired Hedges’ g) distribution is indicated by the grey area (estimated using 10,000 bootstrap iterations), and a 95% confidence interval is illustrated by a straight black line. Enrichment of Gene Ontology (GO) terms (*p* < 0.05) for WTD-fed mice is shown in panel (**D**). Orange dots refer to terms enriched in knockout mice, blue to enriched terms in controls. Combined enrichment scores (c) are shown on the x-axis (c = log(*p*) × z, where *p* refers to the *p*-value from Fisher exact test and z is the z-score computed by assessing the deviation from the expected rank).

To assess the potential metagenomic effects of diet and genotype, we used PICRUSt2 to infer gene identity from the 16S data. After clr-transformation of the predicted KEGG identifiers, we found an effect of diet (PERMANOVA, *p* = 4.3 × 10^−4^, R^2^ = 0.1459) but not genotype (PERMANOVA, *p* = 0.1373, R^2^ = 0.0680), and no interaction between diet and genotype (*p* = 0.6745, R^2^ = 0.0326). This finding is consistent with the findings of the 16S analysis shown above. The effects of diet and genotype were further analyzed using a linear regression model against the first six principal coordinates (PC1–PC6, which explained 73.3% of the total variance), and we found that PC2 strongly correlated with diet (*p* = 4.9 × 10^−5^) and that PC5 tended to be affected by diet (*p* = 0.0566) (Figure 4A).

The effect of diet was further examined to identify differential abundant KEGG identifiers in WTD- and chow-fed mice using ALDEx2. In total 107 KEGG identifiers were upregulated and 77 downregulated in WTD-fed mice (*p*_adj_ < 0.05, estimated absolute effect size > 0.8). Enrichment analysis of up- and downregulated KEGG identifiers against the GO Biological Process database identified immune response (GO:0033006), regulation of leukocyte degranulation (GO:0043300) and tyrosine phosphorylation of STAT protein (GO:0007260) as upregulated GO-terms in WTD mice, whereas terms related to virus response as downregulated (Figure S2).

To investigate the potential effects of genotype, independently of diet, data from the mice fed the WTD were analyzed. Differential abundance analysis showed 34 upregulated and 68 downregulated KEGG identifiers in the KO vs. the control mice (estimated absolute effect size > 0.8). Enrichment analysis against the GO Biological Process database of differentially abundant KEGG identifiers revealed (t)RNA-splicing to be upregulated in the KO mice, whereas protein repair and nucleotide-related metabolic processes were downregulated (*p* < 0.05; Figure 4D). Differentially abundant KEGG identifiers were further annotated for KEGG pathways, and arginine-related pathways were selected to estimate the size of the genotype effect. The KO mice showed a lower abundance of arginine-related pathways (Hedge’s g = −1.3; Figure 4B,C).

### 3.5. Network Analysis of the Expression Profiles

**Figure 5:**
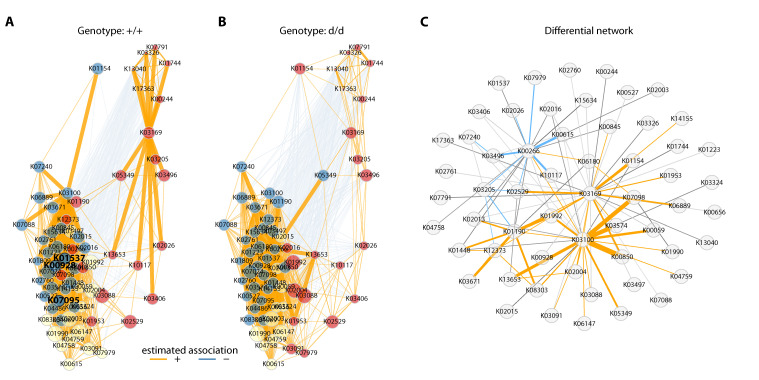
Network analysis of functional profiles. SparCC networks of clr transformed KEGG terms (top 100 terms with the highest variation) for both genotypes regardless of their feeding regime are shown in (**A**) for controls and (**B**) for double knockout mice, respectively. Orange lines denote positive associations, blue lines negative associations; strength of correlations is depicted by the thickness of lines. Estimated clusters of KEGG IDs are shown in blue, red, and yellow, and hub nodes are shown in bold font (controls only). The differential network is shown in panel (**C**), with opposite associations marked in blue (positive in controls and negative in double knockouts) and orange (negative in controls and positive in double knockouts).

Bacteria and their metabolic products do not act as single entities in the gut or body. We aimed to identify differences in the interaction network between control and DKO mice, regardless of the diet fed. To this end, we inferred a correlation network from compositional data, using the top 100 KEGG identifiers with the highest variance according to the PICRUSt2 predictions using SparCC, and significant edges were selected using Student’s *t*-test (*p* < 0.05). The resulting networks for control and DKO mice showed no differences in community modularity (cluster) (three in both groups), but the clustering between the two genotypes significantly differed (adjusted Rand index = 0.349; two-tailed *t*-test *p* < 0.001). Furthermore, in the control mice, three hub nodes (K00928: aspartate kinase, K01537: P-type Ca2+ transporter type 2C, and K07095: uncharacterized protein) were identified, whereas the DKO mouse network had no hub nodes. The global network properties (clustering coefficient, modularity, mean path length, vertex, and edge connectivity) were similar for the genotypes (*p* > 0.05), but the centrality of the two graphs, based on the shortest paths (betweenness centrality), was found to significantly differ (*p* = 0.0351) (Figure 5A,B). On the basis of the permutations (100,000 bootstraps), we constructed a differential network, and significantly correlated node pairs were selected (adaptive Benjamini-Hochberg corrected *p* < 0.01) that showed differentially associated KEGG identifiers for the control and DKO mice. Interestingly, more KEGG identifiers were positively associated with DKO than control mice (Figure 5C).

## 4. Discussion

In addition to the conventional risk factors for CAD, recent studies have identified the contribution of gut bacteria to its etiology and pathogenesis [25,48,49,50]. Indeed, gut dysbiosis appears to be involved in the pathogenesis of many metabolic diseases, including obesity and cardiovascular diseases [18,19,20,21,22]. Furthermore, a diet rich in fat is known to alter the gut microbiome, which to some extent mediates such a diet’s obesogenic effect [51], and a recent study showed an association between the gut microbiota and the consumption of an atherogenic diet by mice [52]. In this context, we bred *Cyp17a1*-deficient mice on an atherogenic *ApoE* KO genetic background to study the role of this gene and a WTD on lipid metabolism and obesity [16]. In our recent study, we showed that CYP17A1 is associated with an obesity and atherosclerosis phenotype [16], but the influence of CYP17A1 on the gut bacteria and the consequences of such interaction for obesity were unclear. Therefore, in the present study, we aimed to determine the effect of diet and *Cyp17a1*-deficiency on the gut microbiota and to relate the findings to the obesity phenotype in these mice.

WTD-feeding was associated with an increase in the number of bacteria belonging to the phylum Firmicutes, independent of the *Cyp17a1* genotype of the mice. This finding is consistent with that of a very recent study that provided compelling evidence that Firmicutes bacteria are enriched in atherosclerotic *ApoE* KO mice consuming a Western diet [52]. Furthermore, the larger number of Firmicutes was reported to be associated with a greater generation of metabolic endotoxins, which cause chronic inflammation [53]. However, further studies are required to identify a causal link between the abundance of key microbial taxa and metabolic endpoints, which might involve gut microbial transplantation into germ-free mice, an established method in mice and other species.

Pathway enrichment analysis revealed that *Cyp17a1* KO mice have a lower abundance of arginine-related pathways. The role of L-arginine in cardiovascular disease is well established; however, the identified effects are in the opposite direction to those that might have been expected from a therapeutic point of view. Several studies have shown a protective effect of L-arginine supplementation against atherosclerosis in animal models of hypercholesterolemia, which is mediated via a nitric oxide (NO)-dependent pathway [54,55,56]. However, Blum et al. recently reported that oral L-arginine therapy does not improve NO bioavailability in patients with CAD and thus may not be beneficial [57]. Therefore, it is still unclear how the arginine pathway relates to cardiovascular disease. In addition, McKnight et al. showed a beneficial role of L-arginine to reduce obesity, increase muscle mass, and improve the metabolic profile of animals and humans [58]. Therefore, the precise role of L-arginine remains uncertain, but it would be interesting to further investigate the role of the L-arginine pathway in cardiovascular disease and obesity.

Network analysis of the expression data identified aspartate kinase as hub node. Aspartate is a common metabolite in pathways that are altered during the development of atherosclerosis [59]. Aspartate is a dicarboxylic amino acid that is involved in the inhibition of fatty streak formation and the progression of atherogenesis [59,60], and has a protective role. In addition, oral supplementation of aspartate is also beneficial for hypertension. Studies conducted in a salt-induced rat model of hypertension demonstrated a reduction in mean arterial pressure of 13 mmHg in aspartate-supplemented animals [61]. Interestingly, the increase in the circulating aspartate concentration that follows its oral administration is accompanied by a rise in L-arginine concentration. Therefore, our network analysis is highly consistent with the results of the pathway enrichment analysis and implies a role for *Cyp17a1* in the regulation of the aspartate/L-arginine axis. However, further studies are needed to clarify the role of the aspartate/L-arginine axis in obesity and body fat distribution, and in particular, to determine whether oral supplementation with either L-arginine or aspartate may reduce fat deposition in the *Cyp17a1* KO mouse model.

## 5. Conclusions and Limitations

Taken together, we further validated the effect of the diet on body mass and composition of their gut microbiome. Using pathway enrichment and network analyses, we could show a substantial impact of Cyp17a1 genotype involving aspartate and L-arginine pathway.

The sample size of this study is considered limited because group sizes are rather small (*n* = 5–6). In addition, from the 16S data, it is not possible to obtain functional profiles (e.g., KEGG identifiers) directly, but one can infer them (as we did, using PICRUSt2) with some uncertainty.

## Data Availability

The data presented in this study are available in Supplementary Material or on request from the corresponding author.

## Supplementary Materials

The following are available online at https://www.mdpi.com/article/10.3390/cells10061292/s1, Table S1. Summary table showing genotype, diet, age, bodyweight, fat, and total cholesterol for each mouse. Table S2. Significant phyla and genera identified using LEfSe (LDA > 2, *p_adj_* < 0.05). Table S3. Phyla, families, and genera tested for differential abundance between conditions. Effect of change (W) and adjusted *p*-values (q) are reported for each comparison.
